# Morphological and Electrical Characterization of MWCNT Papers and Pellets

**DOI:** 10.6028/jres.120.019

**Published:** 2015-12-14

**Authors:** Elisabeth Mansfield, Ari Feldman, Ann N. Chiaramonti, John Lehman, Alexandra E. Curtin

**Affiliations:** National Institute of Standards and Technology, Boulder, CO 80305

**Keywords:** buckypaper, nanotechnology, multiwall carbon nanotubes

## Abstract

Six types of commercially available multiwall carbon nanotube soot were obtained and prepared into buckypapers by pellet pressing and by filtration into a paper. These samples were evaluated with respect to thickness, compressibility and electrical conductivity. DC conductivity results by two-point and four-point (van der Pauw) measurement methods as a function of preparation parameters are presented. Topology was investigated qualitatively by way of scanning electron microscopy and helium ion microscopy and from this, some generalizations about the nanotube structural properties and manufacturing technique with respect to conductivity are given.

## 1. Introduction

Multiwall carbon nanotubes (MWCNTs) play a role in the emerging nanotechnology industry due to the combination of desirable mechanical, thermal, and electrical properties [1]. The global production of MWCNTs is increasing steadily where large nanotube distributers have manufacturing capabilities of 360,000 kg/yr to 450,000 kg/yr. To date, more than 35 commercial products incorporate MWCNTs [2]. At present, bulk MWCNTs serve as a specialty additive for composites and have applications in industries, such as sporting goods, automobile body panels and anti-static coatings. In many MWCNT composites, MWCNTs are used in the form of buckypaper, a dense mat of purified MWCNTs.

The intrinsic properties of buckypaper make it a material considered for use in ways that leverage both electrical and mechanical properties. Properties such as high surface area and electrical conductivity are well suited for charge storage applications, such as batteries and capacitors. Supercapacitors have been enhanced using buckypaper as the electrodes by providing very high conductivity and large surface area [3]. Lithium-ion batteries have shown an increase in storage capacity and potential reduction in size by replacing the graphite anode with high surface area buckypaper [4]. Buckypaper has also been added between polymer layers in composites to add electromagnetic interference (EMI) shielding to cases and equipment [5]. Due to its high thermal conductivity, buckypaper may have a potential application in heat transfer for heat sinks. Finally, high mechanical strength and low density offers the ability for buckypaper to be used for armor applications and structural stability. While buckypaper has been adopted in some products, in order to make the transition from a novel concept to a mass-marketable product, a comparable metric is necessary. A standard reference material based on standardized methods of production and electrical conductivity measurement would be beneficial. This work describes initial steps towards development of such a reference material.

Buckypaper may be prepared in a multitude of specialized processes, but the three-step process developed by Rinzler [6] has been adopted as the standard for both SWCNTs and MWCNTs. First, the carbon nanotubes are dispersed in a surfactant and emulsified to create a CNT colloid. The CNT colloid is then deposited on a filter creating a sheet of CNTs and baked to remove the surfactant. Lastly, the CNT sheet is removed from the filter to create a free-standing buckypaper. While the process is fairly straightforward, details such as the surfactant used, uniformity across the filter, and removal of the sheet from the filter create challenges with respect to reproducible morphology. Additionally, it is possible that any remaining surfactant could increase the electrical resistance and create unstable regions within the film, affecting both the mechanical and electronic properties.

The other technique of interest is traditional ceramic-die pressing, which offers the benefit of producing samples having calculable density and known composition. Die pressing is accomplished by compressing bulk MWCNTs into a cavity to form a pellet. For CNTs, die pressing may fracture the longitudinal structure, which may adversely affect the electrical conductivity [7] yet the ability to reproduce the morphological density is attractive.

Here, the production and evaluation of buckypapers was evaluated for six different MWCNT materials. The goal of this research is to evaluate the quality and consistencies of buckypapers produced from a variety of MWCNT starting materials and provide a comparison between different MWCNT starting materials. The MWCNTs were characterized for composition before formation of the paper, and the buckypapers were qualitatively examined after production. The DC electrical properties of the buckypaper are measured. The impact of the raw soot from which the bulk material is derived and the morphology of the pellets on electrical properties of the final product is discussed.

## 2. Experimental

### 2.1 Materials and Purity^1^

Six different MWCNT materials were selected from commercial manufacturers and are denoted Materials A – F. All materials are from manufacturers with large production capabilities. The materials were characterized for purity using thermogravimetric analysis (TGA). Samples were heated at a rate of 10 °C/min to 1000 °C with 10 mL/min air flow. Three samples of each material were decomposed and the average compositional information (± 1 s.d. or standard deviation) from TGA, along with manufacturer-reported lengths are given in Table 1. Residual mass at 150 °C was used as a measure of water content and residual mass at 800 °C was used as the ash content of the material. In the cases where a negative residual mass is reported, the measurement is within error of zero, and the material was considered to have no contaminates stable to 800 °C. No amorphous carbon was observed for any sample by TGA.

### 2.2 Buckypaper Preparation

Two methods were used to prepare the MWCNT buckypapers. The resulting buckypapers will be referred to as “pellet” for die-pressed material or “filtered” for the filter derived paper throughout this work.

#### 2.2.1 Pellet Formation

A stainless steel pellet die was manufactured to prepare buckypaper pellets [8]. Images of the pressed pellets can be seen in Supplementary Information Fig. 1. The custom die differs from a traditional die by having a central cavity lined with a glass-nylon insulator so that electrical measurements may be made directly on the contents of the die. Bulk MWCNT material was loaded into the fixed volume die, weighed to account for the mass and subsequent density of the raw material, and then pressed at varying pressures (690 kPa, 860 kPa, 1035 kPa, 1200 kPa) by means of a manual press. Assuming a fixed volume of material, the change in length of the die was measured to determine the thickness of the paper formed as well as to compare compressibility of the MWCNT materials.

#### 2.2.2 Traditional Filtered Papers

Bulk MWCNT materials were sent to a commercial manufacturer to be prepared in a process similar to the filtration method developed by Rinzler et al. [6]. All samples were ultrasonicatically agitated in 1700 mL in filtered cold tap water with 5 drops of Triton X-114 surfactant with a Hielscher 400s ultrasonicator equipped with a H14 sonotrode operated at 45 % amplitude and 0.7 duty cycle. Sonication time was approximately 1 hour – 2 hours and varied for each paper based on the time needed to maximize the dispersion of MWCNTs in suspension. In conjunction with sonicating, an IKA RW20 electric mixer was used at 690 rpm – 715 rpm. The samples were filtered through a batch filtration apparatus and allowed to dry at room temperature under a weight. Three of the six MWCNT samples yielded successful filtered papers at various areal weights, reported in grams per square meter (gsm). For Material E, areal weights of 20 gsm, 47 gsm, and 72.9 gsm were achieved. For Material F, areal weights of 27 gsm and 89.3 gsm were prepared. The manufacturer provided thicknesses for all buckypapers produced.

### 2.3 Microscopy

Scanning electron microscopy (SEM) images were collected for each raw soot MWCNT material prior to processing into buckpaper form. SEM was performed using a field emission source at an accelerating voltage of 5 kV together with a 30 μm aperture and 5 mm working distance. Images were collected using an in-column-type detector. Helium ion microscopy (HIM) was used to image the surface of both the pressed pellets of carbon nanotube material and the filtered buckypaper. The incident He ion beam generates a secondary electron signal that provides excellent resolution and depth of field for these samples across many levels of magnification. Samples were prepared for imaging by mounting the papers on carbon tape adhered to aluminum stubs and imaged using a scanning ion beam current of 0.2 pA – 0.4 pA and a dwell time of 10 µs. Samples were generally robust under the beam over the time needed to focus and image the samples. For higher magnification imaging (field of view < 800 nm), shorter dwell times were used to avoid damaging the nanotubes with the ion beam.

### 2.4 Conductivity

#### 2.4.1 Two-point DC Measurement

The resistivity measurements of the buckypaper pellets were performed while still under pressure by use of two contact probes attached to the top and bottom die pistons. A source-measure instrument was used to provide a range of test currents between −1 A and 1 A where the slope of the current-voltage plot identifies the bulk resistance. The resistivity (ρ) of the material was calculated using the equation:

$$\rho_{2\mathrm{pt}}=\frac{A\frac{\delta V}{\delta I}}{d}$$ (1)

where $\frac{\delta V}{\delta I}$ is the slope of the current-voltage plot, *A* is the cross-sectional area of the paper, and *d* is the thickness of the paper. The cross-sectional area was defined by the die cavity with a nominal diameter of 13 mm. For these measurements, the paper thickness *d* was calculated by first measuring the total die thickness using calipers with 10 µm resolution, then subtracting out the total length of the die pistons.

### 2.4.2 Four-point DC Measurement

Filtered buckypaper samples of varying areal weight were made to determine how the thickness or density would affect the DC conductivity. For each material at each areal weight, two samples measuring approximately 10 mm × 10 mm were cut from the 160 cm^2^ prepared filtered papers. Samples from opposite sides of the filtered papers were chosen to anticipate variations in processing that may affect the conductivity. A modified Van der Pauw resistivity setup was created to measure the filtered buckypaper [9]. The measurement setup was composed of three printed circuit boards (PCBs), where the bottom board contained the circuitry necessary for the measurement. Spring-loaded probes were positioned along a diagonal hole pattern to accommodate different sample sizes. The circuitry was isolated from the papers with a second PCB containing only the hole pattern. The samples were clamped between the second and top PCB without holes, where spring-loaded probes can make isolated contact with the buckypaper. For van der Pauw measurements, a test current (*I*) was applied for a combination of measurements (*V*1 through *V*8 in Eqs. (2) and (3) between the probes and the resulting average voltage ratio results in the bulk resistance. The resistivity (ρ) was calculated using the set of equations:

$$\rho_{vdp1}=\frac{\pi }{\ln\left(2\right)}f_{a}d\frac{V1-V2+V3-V4}{4I}$$ (2)

$$\rho_{vdp2}=\frac{\pi }{\ln\left(2\right)}f_{b}d\frac{V5-V6+V7-V8}{4I}$$ (3)

where *f_a_* and *f_b_* are geometric variables that for square geometries equal one. To calculate the bulk resistivity of the papers, test currents of 1 mA and 3 mA were applied and the as-provided thickness of the samples were used. Because the direction of the test currents varies between the measurements, the average of resistivities found in Eqs. (2) and (3) was used as the bulk resistivity of the material. For comparison between samples, all provided papers were cut to approximately 1 cm^2^ squares for measurement.

### 2.5 Thickness

#### 2.5.1 Focused Ion Beam (FIB) Thickness Measurement

The thickness of the filtered buckypapers was measured by milling a 30 μm × 20 μm rectangle into the center of the papers using a 16 nA Ga+ beam in a dual-beam FIB/SEM microscope. The Ga+ ion milling was stopped when the beam penetrated the bottom of the paper, as observed by SEM inspection. A thickness measurement could then be made by simple SEM imaging of the resulting cross-section face.

#### 2.5.2 Micrometer Thickness Measurements

The thickness was measured using precision micrometers with a 0.01 mm Vernier graduation. The carbide-tipped measuring faces were 6.5 mm diameter with 0.00076 mm flatness and an accuracy of 0.001 mm.

#### 2.5.3 SEM Thickness Measurement

Papers were cut with a razor blade into approximately 1 cm squares, then gently dropped onto a carbon tape-coated scanning electron microscopy (SEM) sample stub. The thickness of the edge of the paper was measured on each side in turn by tilting the specimens relative to the SEM beam so that they could be viewed in a projected cross-section. The actual thickness of the paper was determined by then correcting the measured value of thickness for the stage tilt angle (relative to the SEM beam).

## 3. Results and Discussion

### 3.1 Bulk MWCNT Properties

Multiwall carbon nanotubes can be produced by a variety of methods, and with highly variable purities. Thermogravimetric analysis has been used to evaluate purity and composition of carbon nanotube soots [10] and provides a good overview of MWCNT material quality here. In all cases, there is little observable mass loss up to the major transition for the oxidation of the multiwall carbon nanotubes. The oxidation temperatures for each material are given in Table 1. Each material had oxidation temperatures in the range expected for multiwall carbon nanotubes [1], without additional shoulders which could indicate other structures (e.g., single-wall carbon nanotubes, graphitic carbons) or potentially bundling of the MWCNTs. The water composition of all the materials, as measured by mass loss up to 150 °C, was consistent (less than 0.3 % RSD in mass loss) and measured to be less than 1 % of the total mass. All decomposition of the carbonaceous material was complete by 800 °C, leaving ash behind composed of metal catalyst particles and their oxidation products. For all MWCNT samples, the material did not continue to decompose when heated from 800 °C to 1000 °C, thus ash content was measured at 800 °C. The residual mass (% of total mass) of the material at 800 °C is often indicative of purity. Materials of good quality without a post-production purification process typically have 6 wt % – 8 wt % of the sample that can be attributed to metallic catalyst residue. MWCNTs which are purified after production often contain less than 1 wt % catalyst residue. Manufacturers of Materials A – C likely use a purification process.

### 3.2 Buckypaper Manufacturing

Buckypapers were prepared using two different methods, the pellet method and the traditional filtered-paper method. The pellet method was considered the most representative of the carbon nanotubes alone, as no additives were needed to produce the pellets. For statistical purposes, three pellets at each pressure for each material were produced. Raw MWCNT material was loaded into the stainless steel dies and compressed using a range of pressure values. For simplicity, keeping the raw materials in their as-provided state allowed for equal treatment of all materials. It is possible that additional processing, such as the addition of surfactants, could have increased the possibility of successful pellets, but this was not investigated. While all materials were subjected to the same nominal pressures of 690 kPa, 860 kPa, 1035 kPa, 1200 kPa, pellets with compressibility (Fig. 2) greater than 80 % formed robust pellets. For the range of pressures applied, the materials tended to stay within a narrow band of compressibility. The materials that could not form pellets that stayed together (i.e., robust pellets) were unable to do so even with additional pressure. Only materials C, E, and F formed pellets that maintained structural stability after removal from the dies. With only two dies of the same size, we are unable to assess the minimum material required to maintain structural stability for all materials.

Buckypaper is most commonly made by filtration of a carbon nanotube suspension. Surfactant was necessary to produce the filtered buckypapers to ensure adequate dispersion of the MWCNTs; thus, the final products in this study do contain some surfactant. Because of the difficulties in suspending carbon nanotubes in a consistent manner, filtration only yielded successful buckypapers from Materials C, E, and F. For all other carbon nanotubes, the MWCNTs could not be suspended in a dispersion that yielded a functional paper that could be removed in a sheet. Materials C, E, and F were also the only materials that formed successful robust pellets, while the other materials formed pellets that crumbled when removed from the press or could not be pressed into pellets (Table 2). The success of both methods showed a dependence on the raw soot used and demonstrated that compressibility may factor into how well buckypapers form. Successfully pressed pellets or filtered papers from Materials C, E, and F will be discussed in more detail in further sections.

### 3.3 Microscopy

**Fig. 1:**
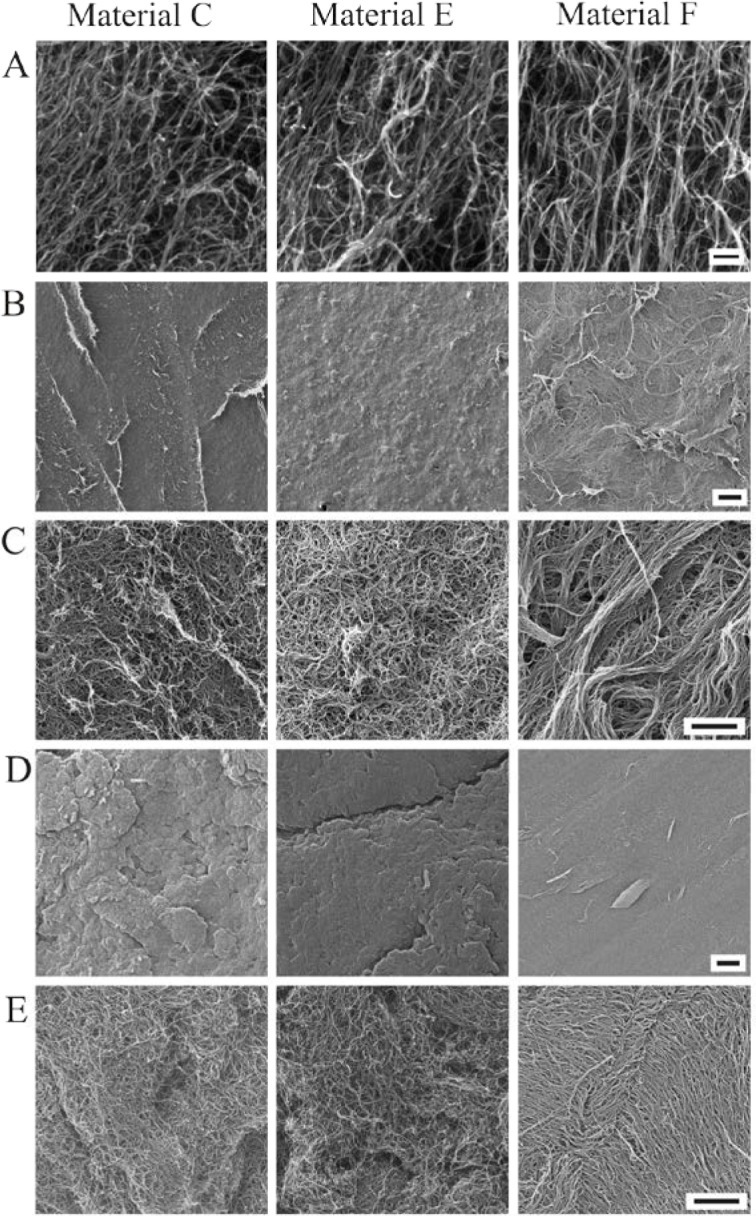
SEM and HIM microscopy of MWCNT buckypapers of Materials C, E, and F. A. Raw MWCNT soot prior to pressing imaged with SEM. Scale bar = 200 nm. B. Filtered buckypaper at 70 gsm or 89 gsm imaged with FIB. Scale bar = 10 µm. C. Close-up of filtered buckypaper at 70 gsm or 89 gsm imaged with FIB. Scale bar 1 µm. D. Pressed pellet buckypapers at 1200 kPa imaged with FIB. Scale bar = 10 µm. E. Close-up of pressed pellet buckypapers at 1200 kPa imaged with FIB. Scale bar = 1 µm.

**Fig. 2:**
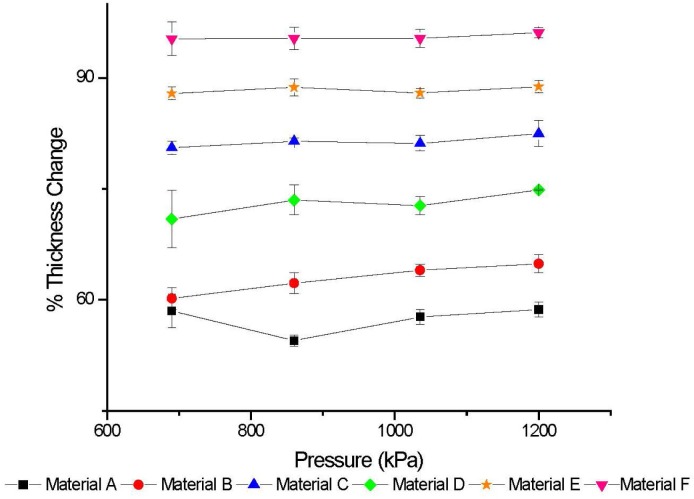
Compressibility of buckypaper pellets. The % thickness change is reported with error bars that are ± 1 standard deviation.

Buckypapers must have well-connected, interwoven networks of carbon nanotubes in order to achieve the desired electronic properties in many composites. SEM and HIM imaging were used to evaluate the quality of the produced buckypapers. Images of the three MWCNT materials that yielded buckypapers can be seen in Fig. 1A. Multiwall structures were evident with few catalyst particles. Material F, which is manufactured as a vertically aligned forest on a surface that was then flattened down has well-aligned nanotubes. Materials C and E were are not aligned in any way.

The filtered papers for Materials C, E, and F were imaged after production at 70 gsm or 89 gsm at low magnification (Fig. 1B). For Material E, the surface was a fairly smooth surface with no noticeable defects. Material C had large ridges evident in the macroscopic view. Material F had the most variability in the surface, with large ridges evident throughout the paper. Defects of this nature could impact the conductivity of the paper.

Further evidence into the structure of these materials is given in Fig. 1C where a close-up view of the filtered papers can be seen. Material C seemed to form ridges, but overall is fairly clearly interwoven MWCNTs. Material E was the most consistent with uniformity throughout and Material F showed long bundled ropes of MWCNTs, most likely due to the highly aligned starting structure.

Defects in the surface of the pressed pellets can be seen in Fig. 1D. Large cracks (Fig. 1D, Material E) were observed in the pressed pellets along with large ridges and clumps (Fig. 1D, Materials C and E). At higher magnifications (Fig. 1E), the pressed pellets appeared more dense than the filtered papers in Fig. 1C, especially in the case of Material F. The junctions between nanotubes in the pressed pellets were expected to be smaller, and this would lead to a higher conductivity over the filtered paper, as the tubes would be able to make closer contact to one another. There is no evidence to support that there were more fractured carbon nanotubes in the pressed pellets than the filtered papers.

### 3.4 Thickness Measurements

One of the difficult issues with regard to buckypaper is producing a paper with a consistent thickness across the bulk of the paper, which appears to have variations on the micrometer scale. Because the thickness is used to calculate the conductivity of the paper and can have an impact on overall effectiveness in composite materials, accurate thickness measurements are important. Three independent methods were used to measure the thickness of filtered buckypapers, highlighting the difficulty in measuring the thickness in this very non-uniform material. The thickness measurements for Material E at 20 gsm are presented in Table 3. In general, micrometer measurements of buckypaper thickness are the least precise as there is not enough physical resolution on the micrometer spindle to evaluate fine features and the thickest area is determined in the measurement. Further, the measurements are necessarily an average over the size of the anvil. Due to such inherent limitations of the method, the measured thickness using precision micrometers should be considered the upper bound for the thickness of the piece examined, and is not representative of the subtle variation in topography of the sample. SEM edge measurements have higher spatial resolution and can show some micrometer-scale variation due to the topography; however, they are extremely dependent on how the specimens are prepared for microscopy. Often, cutting induces compression or bending of the paper edge, and will directly affect the measured thickness. Finally, FIB-cut cross sections are the most accurate measurement of film thickness at the single point at which they are created, but only represent the thickness at a single point in space.

### 3.5 Conductivity Measurements

#### 3.5.1 Two-Point DC Conductivity

**Fig. 3:**
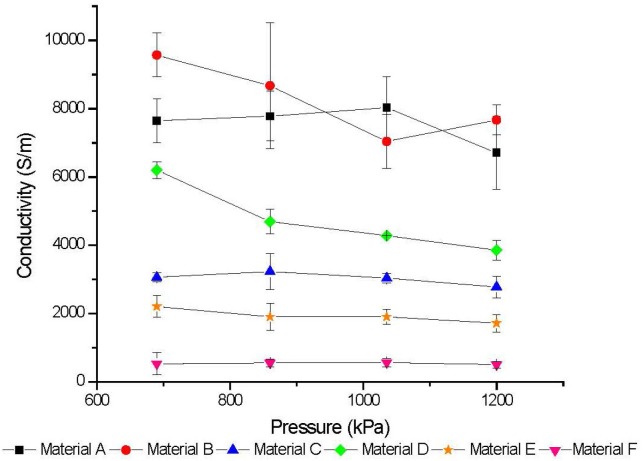
Two-Point Conductivity of Buckypaper Pellets. Error bars represent 1 standard deviation.

Bulk conductivity relies on the thickness, which in the case of some materials was difficult to determine. In some instances, the pressed pellets bowed and/or disintegrated upon removal from the die. To ensure that the thickness and density were fixed during measurement of the conductivity, measurements of the pellets were acquired while the die was still under pressure. A current range between −1 A and +1 A was applied and the resulting voltage was measured. The slope of the measured current-voltage relationship was the resistance and the resistivity was calculated by Eq. (1). For all samples, the current scan was run increasing and decreasing the current over the range +/− 1 A to determine if there was hysteresis due to the scan direction, and the average of the two scans was calculated. The data for all materials with respect to the pressure applied are presented in Fig. 3.

Overall, the materials that formed more robust papers after extraction (C, E, and F) exhibited lower conductivity than the ones that did not form papers at all. The link between conductivity and composition or morphology was difficult to interpret for each material with this measurement. While a definitive correlation between individual material properties and conductivity would be desirable, this relationship is a superposition of the nanotube length, orientation, composition, number of walls, and packing density. It can be said that for each material there was a distinct range of conductivity that generally decreased with increasing density (Fig. 3). The decreasing thickness of the papers is taken into account in the calculation so this could be linked to MWCNT damage during pressing despite the previously imaged surface morphology indicating no obvious widespread damage. It is also interesting to note that material F is made from vertically-aligned nanotubes and exhibits the lowest conductivity. The vertically-aligned nanotubes lay flat after pressing, and it is possible the alignment could lead to better conductivity in the planar direction of the tube, not in the out-of-plane direction of testing.

### 3.5.2 Four-Point DC Conductivity

Each sample was measured with the custom van der Pauw setup using 1 mA and 3 mA test currents. For each measurement, 50 voltage readings were acquired for statistical purposes. Using the thickness provided by the manufacturer and the measured voltages, the conductivity was calculated using Eqs. (2) and (3) (Table 4). For the 4-point measurement, the average conductivity and standard deviation is a function of eight measurement configurations among the same sample, whereas for the 2-point measurement, the statistics reported are for multiple samples.

In contrast to the 2-point conductivity measurement on the pressed pellets, filtered buckypaper from Material F exhibits the highest conductivity in the van der Pauw setup where the direction of measurement is in the plane of the paper as opposed to perpendicular to it in the 2-point measurement. The bulk conductivity increased among all of the materials with the largest increase (~100 times) apparent with the paper made from wafer-grown aligned nanotubes. The increased conductivity could be attributable to the more gentle nature of the filtered processing, preserving the nanotube structure, or due to the direction of measurement. It is difficult to deconvolve the source of the increased conductivity because the conductivity measurements are inherently different. Regardless, despite changes in areal weight the bulk conductivity for each material stays within a range unique to each material. For all of the materials that produced papers, the measured values here fit between previously reported values of amorphous carbon (1 × 10^2^ S/m) [11] and graphite (2.0 × 10^5^ S/m). [12]

## 4. Conclusion

Six bulk MWCNT materials were characterized for purity and composition before being processed into both pellet and filtered buckypapers. The goal of this research was to evaluate the quality and consistencies of buckypapers produced from a variety of MWCNT starting materials. All MWCNT materials tested had oxidation temperatures in the range of 600 °C – 700 °C, which would be expected for a material consisting of mainly MWCNTs with very little water content. Materials C, E, and F yielded successful buckypapers and pellets. With the exception of Material C, all post-production purified materials (ash content of < 6 %) are not compressible and do not yield buckypaper through the pellet or filtration methods. The unpurified materials, Materials E and F, have residual mass on the order of 7 % and can be made into both pellet and filtered papers.

The 2-point and 4-point conductivity measurements yielded similar results for Materials C and E, which had comparable conductivities. These materials were found to have similar morphologies when the buckypapers and pressed pellets were imaged. Material F exhibited some unique conductivity behavior by demonstrating the lowest 2-point conductivity and highest 4-point conductivity due in part to the highly ordered structure of the carbon nanotubes. Moreover, the variability of thickness measurements demonstrates that calculating the bulk conductivity is not trivial, and measured values should be considered based on average thicknesses measured by FIB cross-section over as many points as realistically possible. Overall, production of buckypapers is highly variable and are dependent on starting material.

## Figures and Tables

**Fig. 4:**
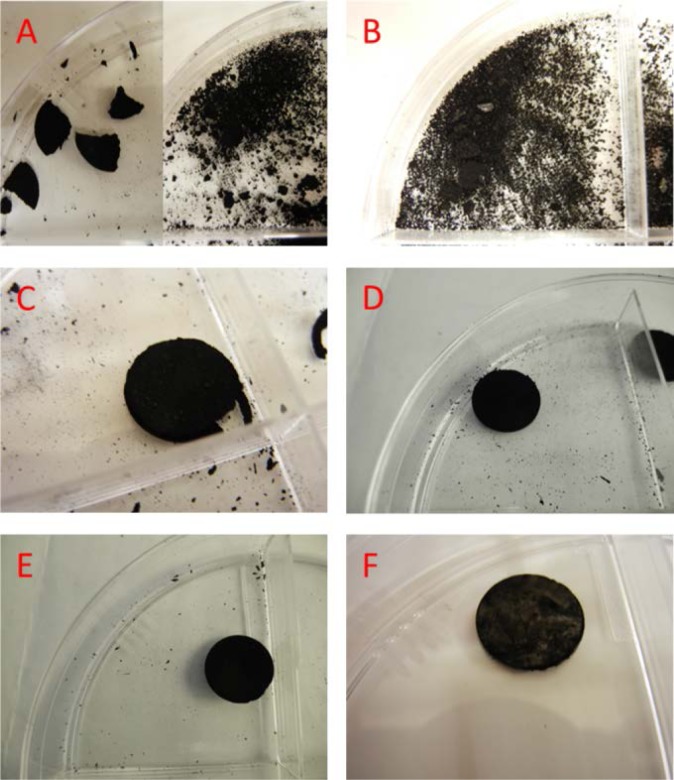
In conjunction with the brief results presented in Table 2, a visualization of the pellet formation assists in understanding the successes and failures of each MWCNT material. All pictures in this supplementary information represent the average pellet produced with 690 kPa of pressure. Two different samples of Material A produced mixed results of no pellets at all and brittle chunks of pellets. Neither type of result was considered a successful paper (i.e., one that can be handled reasonably without falling apart) nor no amount of pressure changed the results shown. The raw Material B can be described as sandy and gritty, thus when CNT pellets did not form under pressure, only small grain-like chunks remained. Materials C–E produced pellets of varying durability. In the image, Material C can be seen crumbling along the edges, while Materials D and E are solid. Material F was produced from the vertically-aligned CNT raw material and the resultant pellets were the thinnest and most pliable. It is interesting note that even with the macroscopic view of this material, the phase domains of the aligned nanotubes are visible by changes in the reflectivity.

**Table 1:** MWCNT Composition Information

Material	Residual Mass % at T (°C)				Oxidation T		
	150	±1 s.d.	800	±1 s.d.	Tox (°C)	±1 s.d.	Reported Length
A	99.63	0.15	1.03	0.51	675.87	6.05	10 µm
B	99.82	0.04	−0.12	0.10	679.37	9.74	3 µm
C	99.63	0.31	−0.68	0.77	661.63	11.74	> 1 µm
D	99.58	0.34	7.02	0.90	643.10	4.33	0.1 – 10 µm
E	99.40	0.18	6.51	1.09	657.23	11.23	1.5 µm
F	98.05	0.63	−0.14	0.67	737.93	8.82	Not reported

**Table 2:** Buckypaper yield as a function of MWCNT

Material #	Pellet	Filtered
A	Inconsistent results	No paper
B	Crumbles, not durable	No paper
C	Thin robust pellet	20, 54, and 70 gsm
D	Thin robust pellet	No paper
E	Thin, dense, robust pellet	20, 47, 70 gsm
F	Ex. Thin, Bendable pellet	27, 89 gsm

**Table 3:** Thickness measurement for Material E 20 gsm paper measured by three methods

Method Used	Measured Thickness (µm) ± 1 s.d.
Micrometers: 16 measurements	50 ± 0
SEM: 2 different edges, 12 measurements	33.97 ± 3.59; 24.52 ± 2.89
FIB Cross Section: 2 measurements	39.26 ± 0.13

**Table 4:** Four-point probe measurements of conductivity on filtered buckypaper

Material	Areal Weight (gsm)	Position	Conductivity (S/m) ± 1 s.d.
C	20	1	6.02 × 10^3^ ± 3.7
	20	2	5.91 × 10^3^ ± 3.7
C	54	1	6.22 × 10^3^ ± 15.03
	54	2	6.25 × 10^3^ ± 14.15
C	70	1	6.37 × 10^3^ ± 15.93
	70	2	6.35 × 10^3^ ± 16.67

E	20	1	7.24 × 10^3^ ± 3.39
	20	2	6.32 × 10^3^ ± 2.54
E	54	1	5.92 × 10^3^ ± 11.66
	54	2	5.71 × 10^3^ ± 9.28
E	70	1	5.27 × 10^3^ ± 12.35
	70	2	5.35 × 10^3^ ± 11.67

F	27	1	1.26 × 10^4^ ± 25.75
	27	2	1.34 × 10^4^ ± 27.07
F	89	1	1.96 × 10^4^ ± 396.10
	89	2	1.94 × 10^4^ ± 282.30

## Footnotes

1 Certain commercial equipment, instruments, or materials are identified in this paper to foster understanding. Such identification does not imply recommendation or endorsement by the National Institute of Standards and Technology, nor does it imply that the materials or equipment identified are necessarily the best available for the purpose.
